# The Diagnostic Value of Cervical Lymph Node Metastasis in Head and Neck Squamous Carcinoma by Using Diffusion-Weighted Magnetic Resonance Imaging and Computed Tomography Perfusion

**DOI:** 10.1155/2014/260859

**Published:** 2014-06-24

**Authors:** Jin Zhong, Zonghong Lu, Liang Xu, Longchun Dong, Hui Qiao, Rui Hua, Yi Gong, Zhenxing Liu, Caixian Hao, Xuehuan Liu, Changqing Zong, Li He, Jun Liu

**Affiliations:** Department of Imaging, Tianjin Union Medicine Centre, Tianjin 300121, China

## Abstract

*Purpose.* The aim of this study was to compare diffusion-weighted magnetic resonance imaging (DWI) with computed tomography perfusion (CTP) for preoperative detection of metastases to lymph nodes (LNs) in head and neck squamous cell carcinoma (SCC). *Methods.* Between May 2010 and April 2012, 30 patients with head and neck SCC underwent preoperative DWI and CTP. Two radiologists measured apparent diffusion coefficient (ADC) values and CTP parameters independently. Surgery and histopathologic examinations were performed on all patients. *Results.* On DWI, 65 LNs were detected in 30 patients. The mean ADC value of metastatic nodes was lower than benign nodes and the difference was statistically significant (*P* < 0.05). On CTP images, the mean value in metastatic nodes of blood flow (BF) and blood volume (BV) was higher than that in benign nodes, and mean transit time (MTT) in metastatic nodes was lower than that in benign nodes. There were significant differences in BF and MTT values between metastatic and benign LNs (*P* < 0.05). There were significant differences between the AUCs of DWI and CTP (*Z*=4.612, *P* < 0.001). *Conclusion.* DWI with ADC value measurements may be more accurate than CTP for the preoperative diagnosis of cervical LN metastases.

## 1. Introduction

Squamous cell carcinoma (SCC) is the most common malignancy of the head and neck region. It accounts for 5% of all malignant tumours worldwide [1]. A meta-analysis by Dünne et al. [2] showed a 5-year survival rate between 17% and 55.8% for SCC with cervical node metastases and 44.6–76% for SCC patients without cervical node metastases. The presence of multiple metastatic lymph nodes (LNs) is presumed to be a worse prognostic sign [3]. The detection of cervical node metastases provides very important prognostic information and often helps decide the treatment of head and neck SCC. Node sampling is the definitive method of differentiating benign LNs from metastatic LNs, but biopsy methods are invasive and operator-dependent, with a high incidence of false-negative results [4, 5]. With advancements in imaging methods, several noninvasive imaging techniques have arisen, with the potential for identifying benign and metastatic LNs in head and neck SCC, thus avoiding the complications due to biopsy sampling [6].

Although ultrasound, routine contrast-enhanced computed tomography (CT) and magnetic resonance imaging (MRI) allow the detection of enlarged cervical LNs, none of these methods can distinguish between benign and malignant causes of enlargement with any accuracy [7], as they use standard parameters (shape, size, internal architecture, extranodal invasion, and vascular features) that are nonspecific for malignancy [8, 9].

Single photon emission computed tomography (SPECT) and positron-emission tomography (PET) are imaging techniques that supply functional information, but they involve radiation exposure and are expensive with low availability and are hampered by relatively low spatial resolution [10–13].

Recently, other functional imaging techniques such as MRI diffusion-weighted imaging (DWI) and CT perfusion imaging (CTP) have shown promise in detecting metastatic cervical LNs, and there is increasing experience in head and neck SCC. DWI is an MR technique that depicts molecular diffusion, which is the Brownian motion of water protons in biologic tissues. Examination of molecular diffusion using DWI performs with an EPI (echo planar imaging) sequence and a linear regression after a logarithmic transformation of the signal intensity was used to calculate the ADC values [14]. To date, the diagnosis of LN metastases has been based mainly on size criteria; however, nonenlarged nodes may harbour malignancy, whereas benign reactive nodes may be enlarged. Promising results with DWI to help detect cervical LN metastases (especially in normal-sized nodes) and to differentiate between benign and malignant enlarged nodes have been reported. The general consensus is that ADCs of malignant nodes are significantly lower than those of benign nodes [15].

Unlike conventional contrast-enhanced CT, which is normally assessed visually, perfusion imaging requires quantification of the enhancement in tissue and blood at certain time points following intravenous injection. These enhancement data are used to calculate blood flow (BF), blood volume (BV), and mean transit time (MTT) for each voxel, depicted in a color-coded display. Data processing methods are based on the robust physiological principles of compartmental analysis or linear systems theory. In the compartmental modeling technique, analysis can be done by one compartment method which assumes the intravascular and extravascular compartment as a single compartment and allows measurement of tissue perfusion during the first pass of contrast [15, 16].

Although DWI and CTP are increasingly used to detect LN metastases in head and neck SCC, comparison of the diagnostic value of the two imaging modalities has been rarely performed. The aim of this study was to compare the value of DWI and CTP for detecting metastatic LNs which were confirmed by pathologic diagnosis in patients with head and neck SCC.

## 2. Materials and Methods

### 2.1. Ethics Statement

The study was approved by the local ethics committee and all patients signed review board-approved consent before participation.

### 2.2. Patients

Sixty-five cervical LNs from 30 treatment-naïve patients with head and neck SCC were excised during surgery from May 01, 2010, to April 30, 2012. All LNs underwent pathological analysis. Among the 30 patients, 21 were men and 9 were women; their age ranged from 38 to 70 years, with a mean age of 53.6 years. The primary cancers were of the larynx (*n* = 9), tongue (*n* = 3), nasopharynx (*n* = 6), floor of mouth (*n* = 3), nasal cavity (*n* = 4), oropharynx (*n* = 4), and gingiva (*n* = 1). All patients underwent DWI and CTP before surgery.

### 2.3. Imaging Protocols

#### 2.3.1. MRI/DWI

All MRI examinations were performed using a 1.5 T MRI unit (Philips Intera Achieva, Philips Medical Systems, Best, The Netherlands) with a head and neck coil. Thirty patients underwent conventional MRI and DWI to include nodes from the base of the skull to the suprasternal notch. Before scanning, all patients were trained to avoid swallowing during the MRI examination.

In all patients the following protocol was performed:

- fast spin-echo (FSE) T2-weighted images (TR, 4600 ms; TE, 80 ms; slice thickness, 3 mm) in the axial plane;
- fast spin-echo (FSE) T2-weighted images (TR, 3850 ms; TE, 75 ms; slice thickness, 3 mm), in the coronal plane;
- fast spin-echo (FSE) T1-weighted images, with fat suppression (TR, 480 ms; TE, 15 ms; slice thickness, 3 mm) in the axial plane;
- diffusion-weighted imaging with background body signal suppression (DWIBS) images (TR, 17131 ms; TE, 60 ms; TI, 165 ms; Matrix 132 × 98; SENSE factor 2; NSA, 6;* b*, 600 s/mm^2^) in the axial and coronal planes. Image of black and white reverse image was constructed.

### 2.4. MRI Data Analysis

The ADC values were automatically measured by standard software (Philips Extended MR Workspace, Philips Medical Systems, Best, The Netherlands). The ADC values were obtained by drawing regions of interest (ROIs) around the solid portions of nodes, avoiding necrotic-appearing areas. Two experienced radiologists analysed the results independently. Disagreements (controversy about positive nodes) regarding image findings were resolved with mutual accord.

### 2.5. CT Perfusion

The thirty patients underwent preoperative routine CT and perfusion CT scans using a multidetector 16-slice CT scanner (Philips MX 8000, Philips Medical Systems, Andover MA, USA). Selection of the nodal targets was based on a plain CT scan; nonionic iodinated contrast agent (Ultravist 370, Bayer, Germany) (45 mL, 350 mg I/mL) was injected at a flow rate of 5 mL/s via the antecubital vein with an injector (Liebel-Flarsheim, Cincinnati, OH, USA) for dynamic perfusion CT scanning. The perfusion CT parameters were as follows: 120 kVp, 150 mAs, 16 × 1.5 detector collimation, 3 mm slice thickness, and a scanning speed of 1 s/rotation. Thus, we could evaluate flow perfusion in eight slices, including 24 mm from top to bottom.

### 2.6. CT Perfusion Data Analysis

Choosing the common carotid or internal carotid artery as the input artery and internal jugular vein as the output vein, we obtained time density curves and calculated BF, BV, and MTT of the ROIs with perfusion software (deconvolution arithmetic) from the workstation (Extended Brilliance, Philips Medical Systems, Best, The Netherlands). ROIs again were placed in solid areas of the LNs, avoiding calcified or necrotic-appearing areas. Two experienced radiologists carried out this procedure and the mean values were calculated.

### 2.7. Statistical Analysis

ADC values and BF, BV, and MTT of the LNs were compared using Student's *t*-test. The two imaging techniques were compared using receiver operating characteristic curves (ROC curves). *P* < 0.05 was considered statistically significant. All statistical analyses were performed with the SPSS 17.0 software package.

## 3. Results

### 3.1. DWI and ADC Values

**Figure 1:**
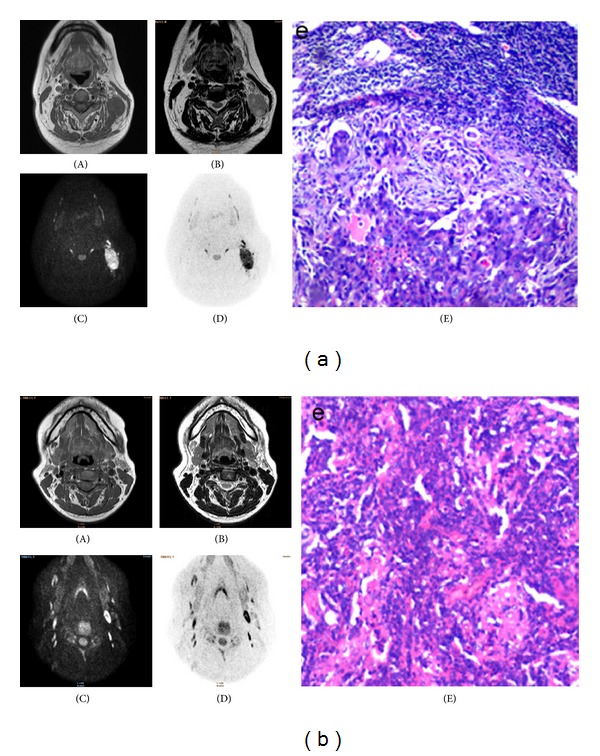
(a): Patient with rhinal cancer. (A) Axial T1-weighted and (B) fast spin-echo-T2-weighted images showing a rounded, enlarged neck node on the left side, showing T1 and T2 signal isointensity. (C) Diffusion-weighted image (DWI) at *b* = 600 s/mm^2^ showed that the node has high signal intensity. (D) On DWI inversion image, the same node exhibits low signal intensity. Mean apparent diffusion coefficient (ADC) value of the lymph node is 0.85 × 10^−3 ^mm^2^/s. (E) Lymph node with metastatic squamous cell carcinoma confirmed by pathologic diagnosis (H&E staining, ×200). (b) Hyperplastic benign lymph node in a case of nasopharyngeal carcinoma. (A) Axial T1-weighted and (B) fast spin-echo-T2-weighted images showing an oval node on the left side, which demonstrates T1 and T2 signal isointensity. (C) Diffusion-weighted image (DWI) at *b* = 600 s/mm^2^ shows that the node has slightly elevated signal intensity. (D) On DWI inversion image, the same node exhibits low signal intensity. The mean apparent diffusion coefficient (ADC) value of the node is 0.85 × 10^−3 ^mm^2^/s. (E) Hyperplastic benign lymph node confirmed by pathologic diagnosis (H&E staining, ×200).

Of the 65 LNs, 48 nodes were proven to be histologically malignant, and 17 nodes were benign. On DWI, 43/48 metastatic LNs showed high signal intensity (*b* = 600 s/mm^2^), whereas on the black and white flip images they presented low signal (Figure 1(a)). Thirteen of the 17 benign nodes were low in signal intensity (*b* = 600 s/mm^2^) on DWI images (Figure 1(b)). The mean ADC value of metastatic nodes was approximately 0.849 × 10^−3 ^mm^2^/s (range: 0.738 × 10^−3^–0.960 × 10^−3 ^mm^2^/s), lower than the mean value of the benign nodes (1.443 × 10^−3 ^mm^2^/s, range: 1.037 × 10^−3 ^mm^2^/s–1.849 × 10^−3 ^mm^2^/s); this difference was statistically significant (*P* < 0.05) with *t* = 2.629 (Table 1). In this study, the best threshold value for diagnosing metastatic nodes was 0.960 × 10^−3 ^mm^2^/s, yielding a sensitivity of 89.58%, specificity of 76.47%, accuracy of 86.15%, PPV of 91.48%, and NPV of 72.22%.

### 3.2. CT Perfusion

**Figure 2:**
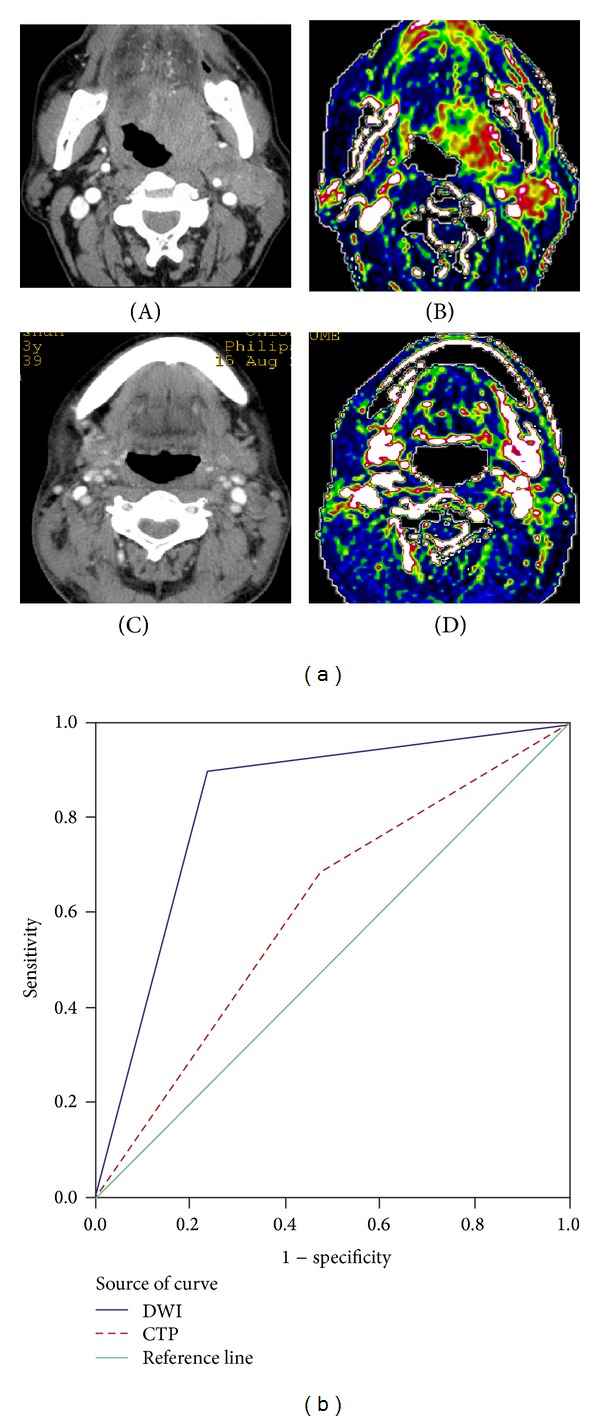
(a) (A-B) Patient with floor of mouth cancer. (A) On contrast-enhanced CT, an intensely enhancing mass is visible in the left base of tongue and left cervical area, with involvement of the sternocleidomastoid. (B) The lesions have high blood flow (BF) values. The mean BF value of the lymph node (LN) is 113.45 mL/100 g/min, (C-D) lymphadenitis with laryngeal carcinoma, (C) enhanced CT showing a nodule in the left cervical area with heterogeneous enhancement. (D) Marginal BF of 86.95 mL/100 g/min. (b) Receiver operating characteristic (ROC) curve for diffusion-weighted imaging (DWI) and CT perfusion (CTP). Areas under the curves (AUCs) are 0.830 and 0.605, respectively. There is a significant difference between the AUC of DWI and the AUC of CTP (*Z* = 4.612, *P* < 0.001).

On CTP images, 33/48 metastatic LNs showed increased perfusion. On conventional enhanced CT, images demonstrated enhancement (Figure 2(a) (A-B)). Nine of 17 benign LNs displayed low blood perfusion and mild-to-moderate enhancement (Figure 2(a) (C-D)) on CTAP and conventional CT images, respectively.

The mean BF, BV, and MTT values for metastatic nodes were 114.62 ± 14.26 mL/100 g/min, 32.15 ± 13.21 mL/100 g, and 5.56 ± 0.39 s, respectively. The mean values for BF, BV, and MTT in benign nodes were 67.82 ± 13.84 mL/100 g/min, 19.36 ± 7.34 mL/100 g, and 9.46 ± 3.23 s, respectively. There were significant differences in BF and MTT values between metastatic and benign LNs (*P* < 0.05) (Table 1). The optimum threshold BF value for differentiating malignant from benign nodes was 100.36 mL/100 g/min, yielding a sensitivity of 68.18%, specificity of 52.94%, accuracy of 64.46%, PPV of 80.48%, and NPV of 37.50%.

Figure 2(b) shows the ROC curves of the ADC and BF values used for differentiating benign from metastatic LNs. The areas under the curve (AUC) were 0.830 and 0.605. There were significant differences between the AUCs of DWI and CTP (*Z* = 4.612, *P* < 0.001) (Table 2) for diagnosing metastatic LNs in head and neck SCC.

## 4. Discussion

### 4.1. DWI and ADC Values

DWI is an MR imaging-based technique whereby diffusion properties of water can be quantified using the ADC. Hypercellular tissue, such as solid tumour, is characterized by a low ADC, while more hypocellular tissue, such as normal tissue, is typically characterized by a higher ADC. As several studies reported, metastatic nodes showed a reduction of diffusivity, which can be attributed to hypercellularity, to an increased nuclear-to-cytoplasmic ratio, and to perfusion [17, 18]. King et al. [19] reported that DWI could improve the accuracy in the distinction between benign and malignant nodes.

Pathological states, such as malignant tumor cell, strongly affect water diffusion more than benign tumor cell and so influence DWI, and hence the technique has attracted considerable research attention for both benign and malignant disease processes.

One of the DWI investigations performed in the head and neck [20] showed the potential of this technique for differentiating benign from malignant lesions. Srinivasan et al. [21] and Abdel Razek et al. [22] reported that there was a significant difference in ADCs between benign and malignant lesions. In one of these investigations [21], the authors found a lack of overlap between the higher ADCs of benign and the lower ADCs of malignant head and neck lesions. Using an ADC obtained with two *b* values (0 and 800 s/mm^2^) and a 3 T MR unit, they established an optimal ADC threshold of 1.3 × 10^−3 ^mm^2^/s for diagnosis. The authors of the other DWI investigation [22] confirmed the difference in ADCs between benign and malignant lesions. In that study, *b* values of 0, 500, and 1000 s/mm^2^ at 1.5 T yielded a significant difference between benign and malignant lesions (*P* < 0.001). Also, an optimal ADC threshold of 1.25 × 10^−3 ^mm^2^/s to help differentiate benign from malignant lesions was established, consistent with that of the previous study [21], and yielded an accuracy of 92.8%, sensitivity of 94.4%, and specificity of 91.2%. Although these results showed the advantage of DWI for diagnosis of head and neck lesions, there will be exceptions and overlap in ADC results. Therefore, a single ADC threshold cannot be used in all conditions, and combining it with specific sites and morphological findings will be necessary.

In our series, the evaluation with DWI showed that metastatic nodes appeared hyperintense (*b* = 600 mm^2^/s); conversely, benign nodes were hypointense (*b* = 600 mm^2^/s). We chose 0.960 × 10^−3 ^mm^2^/s as the optimal ADC threshold value for distinguishing benign from metastatic nodes, with a sensitivity of 89.58%, a specificity of 76.47%, an accuracy of 86.15%, a PPV of 91.48%, and an NPV of 72.22%. Our data are not in agreement with those of Kato et al. [23] who found, for SCC LNs, the mean ADC value (1.45 ± 0.48 × 10^−3^ mm^2^/s) to be higher than that in benign lymphadenopathies (0.89 ± 0.21 × 10^−3^ mm^2^/s).

Differences among these studies can be attributed to several causes. One is the choice of the *b* values: a lower *b* value increases signal-to-noise ratio but lowers the sensitivity to diffusion. Other factors are the size of the ROI (solid portion, necrotic portion, or whole area) and the use of sequences that reduce the artefacts in order to make the measurement of the ROI more precise [24].

### 4.2. CT Perfusion

CTP is a technique that allows quick qualitative and quantitative evaluation of tissue blood perfusion by generating maps of BF, BV, and MTT. Gandhi et al. [25] reported that CT perfusion parameters may provide valid information on angiogenic activity induced by neoplastic cells invading LNs. Tumour vessels in malignant LNs have certain characteristics, including short artery and vein circuits and a lack of smooth muscle around the vessel walls, which can result in increased blood perfusion [26].

In our study, 13/17 metastatic LNs showed high BF and BV and short MTT. There were significant differences between metastatic LNs and benign LNs in BF and MTT. In metastatic nodes, BF is increased by newly developed vessels, while MTT is usually decreased by the presence of pathological arteriovenous shunts. Changes in vascular endothelium and in the function of vessels are induced by neoangiogenesis [27, 28].

In our study, 15/48 metastatic LNs also had lower BF, reflecting the necrotic tumour elements (although any macroscopic nonviable areas were excluded from the analysis), a quite common phenomenon in malignant tumours [29, 30]. This type of inter- or intralesional heterogeneity sometimes resulted in high standard deviations of the perfusion values. This heterogeneity could be a pitfall of measuring perfusion values in ROIs (variable in size and location) scattered through the LNs [26]. It may have been the factor behind the significant differences in BF values, and not in BV values, between benign and metastatic nodes. The best threshold BF value for differentiating malignant from benign nodes was 100.36 mL/100 g/min, yielding a sensitivity of 68.18%, specificity of 52.94%, accuracy of 64.46%, PPV of 80.48%, and NPV of 37.50%.

### 4.3. Comparison between DWI and CT Perfusion

DWI has been reported to be able to distinguish between malignant and benign LNs with sensitivities ranging from 52% to 98% and specificities ranging from 88% to 97% [31, 32]. ADC values played an important role in differentiating between benign and metastatic LNs. Some authors reported similar threshold ADC values such as 0.94 × 10^−3 ^mm^2^/s. Our data was in agreement with the results mentioned above and obtained a sensitivity of 89.58%, a specificity of 76.47%, and an accuracy of 86.15%.

In CTP, there were general differences between benign and metastatic LNs, but with some overlap [33]. We obtained a sensitivity of 68.18%, specificity of 52.94%, and accuracy of 64.46% with BF 100.36 mL/100 g/min as the threshold value. The ROC curve was used to evaluate the two imaging techniques, with an AUC of 0.830 (DWI) and 0.605 for CTP (*P* < 0.001). In addition, the patients undergoing CTP were injected with an iodinated contrast agent that has been confirmed to have some nephrotoxicity; meanwhile, patients inevitably suffer from radiation injury.

## 5. Conclusion

In conclusion, DWI exhibited better sensitivity and specificity than CTP. DWI may be the preferred technique for the preoperative assessment of LNs in head and neck SCC.

## Figures and Tables

**Table 1:** Quantitative measurements of benign and metastatic LNs.

	Benign *n* = 17	Metastatic *n* = 48	*t*	*P*
ADC (×10^−3^ mm^2^/s)	1.443 ± 0.406	0.849 ± 0.111	2.629	0.011
BF (mL/100 g/min)	67.82 ± 13.84	114.62 ± 14.26	3.336	0.002
BV (mL/100 g)	19.36 ± 7.34	32.15 ± 13.21	1.006	0.209
MTT (s)	9.46 ± 3.23	5.56 ± 0.39	2.346	0.002

ADC = apparent diffusion coefficient, BF = blood flow, BV = blood volume, and MTT = mean transit time.

**Table 2:** Accuracy of diffusion-weighted imaging (DWI) and perfusion CT (CTP).

	Sensitivity	Specificity	PPV	NPV	Accuracy	AUC
DWI	43/48 (89.58%)	13/17 (76.47%)	43/47 (91.49%)	13/18 (72.22%)	56/65 (86.15%)	0.830
CTP	33/48 (68.75%)	9/17 (52.94%)	33/41 (80.48%)	9/24 (37.50%)	42/65 (64.61%)	0.605

*Z* = 4.612, *P* < 0.001.

PPV = positive predictive value, NPV = negative predictive value, and AUC = area under the ROC curve.

## Abbreviations

DWI: Diffusion-weighted magnetic resonance imaging
CTP: Computed tomography perfusion
LN: Lymph node
SCC: Squamous cell carcinoma
ADC: Apparent diffusion coefficient
PPV: Positive predictive value
NPV: Negative predictive value.
